# Adult Height after Growth Hormone Treatment at Pubertal Onset in Short Adolescents Born Small for Gestational Age: Results from a Belgian Registry-Based Study

**DOI:** 10.1155/2018/6421243

**Published:** 2018-04-03

**Authors:** M. Thomas, D. Beckers, C. Brachet, H. Dotremont, M.-C. Lebrethon, P. Lysy, G. Massa, N. Reynaert, R. Rooman, S. van der Straaten, M. Roelants, J. De Schepper

**Affiliations:** ^1^The Belgian Society for Pediatric Endocrinology and Diabetology (BESPEED), Bruxelles, Belgium; ^2^Division of Pediatric Endocrinology, Université catholique de Louvain, CHU UCL Namur, Yvoir, Belgium; ^3^Division of Pediatric Endocrinology, Hôpital Universitaire des Enfants Reine Fabiola (HUDERF), Bruxelles, Belgium; ^4^Division of Pediatric Endocrinology, UZ Antwerpen, Antwerpen, Belgium; ^5^Division of Pediatric Endocrinology, CHU-Notre-Dame des Bruyères, Chênée, Belgium; ^6^Division of Pediatric Endocrinology, Cliniques Universitaires Saint-Luc, Bruxelles, Belgium; ^7^Division of Pediatric Endocrinology, Jessa Ziekenhuis, Hasselt, Belgium; ^8^Division of Pediatric Endocrinology, UZ Leuven, Leuven, Belgium; ^9^Division of Pediatric Endocrinology, UZ Gent, Gent, Belgium; ^10^Department of Public Health and Primary Care, KU Leuven, Leuven, Belgium; ^11^Division of Pediatric Endocrinology, UZ Brussel, Brussel, Belgium

## Abstract

**Objectives:**

Information on the efficacy of GH treatment in short SGA children starting their treatment in adolescence is limited. Therefore, adult height (AH), total height gain, and pubertal height gain were evaluated in short SGA children who started GH treatment at pubertal onset.

**Patient and Methods:**

Growth data of 47 short SGA adolescents (22 boys) who started GH treatment at pubertal onset (PUB group) were compared with results from 27 short SGA patients (11 boys) who started GH therapy at least 1 year before pubertal onset (PrePUB group).

**Results:**

The PUB group achieved a mean (±SD) total height gain of 0.8 ± 0.7 SDS and an AH of −2.5 ± 0.7 SDS after 4.1 ± 1.1 years of GH treatment with a dosage of 41.8 ± 8.4 *μ*g/kg/day. These results were comparable with those in the PrePUB group, which was treated for a longer duration (5.8 ± 2.1 years), resulting in a total height gain of 1.1 ± 0.7 SDS and an AH of −2.1 ± 1.0 SDS. Multiple regression analysis showed a significantly lower height gain in pubertal patients, females, and patients weighing less at start of GH treatment. An AH above −2 SDS and above the parent-specific lower limit of height was, respectively, reached in 28% and 70% of PUB and 44% and 67% of PrePUB patients (NS). AH SDS was positively correlated with the height SDS at start of GH.

**Conclusions:**

Short SGA adolescents starting GH therapy at an early pubertal stage have a modest and variable height gain. A normal AH can be expected in one third of the patients, especially in those with a smaller height deficit at onset of GH treatment.

## 1. Introduction

In Europe, growth hormone (GH) treatment is, since 2003, an approved growth-promoting therapy for children born small for gestational age (SGA) who do not show postnatal spontaneous catch-up growth. In Belgium, GH treatment is reimbursed since 2004 for short (<−2.5 SDS) SGA children, aged 4 years or older with a height > 1 SDS below midparental height and without catch-up growth (height velocity (HV) < 0.0 SDS). Adult height (AH) in short SGA children treated with GH is mainly dependent on the duration of treatment: the best response is obtained when treatment is started several years before the onset of puberty [1, 2]. Early diagnosis and referral for treatment of SGA children without catch-up growth before puberty has therefore been advocated [3].

Currently, median age at start of GH treatment in short SGA children in Belgium is 7.7 years (data on file, Belgian Society for Pediatric Endocrinology and Diabetology (BESPEED)). Despite efforts to promote early referral of short SGA children during the last decennium in most European countries, a variable percentage of short SGA children still consults for growth-promoting therapy around the onset of puberty (up to 17% in Belgium). Upper limits for chronological age or bone age for efficacious initiation of GH therapy have not been studied. Increasing the dose of GH and/or additional treatment with GnRH agonists remain controversial issues in the management of short SGA adolescents, presenting with a major height deficit, and are not commonly performed in Belgium [4, 5].

To determine whether it is justified to start a GH treatment at early pubertal onset in short SGA adolescents, we retrospectively analysed our national GH registry. Pubertal height gain and AH were analysed in children who started GH around the onset of puberty and compared with the outcomes in short SGA children who initiated GH therapy at least one year before the onset of puberty. Efficacy of the treatment was measured by the following parameters: mean total height gain (in SDS), mean AH SDS and percentage of patients reaching an adult height > −2 SDS (167.6 cm for boys and 154.7 cm for girls in Belgium), and mean AH SDS corrected for midparental height and percentage of patients reaching an AH above the parent-specific lower limit of height.

## 2. Patients and Methods

### 2.1. Patients

Growth data of GH-treated short SGA children were retrieved from the Belgian registry of GH-treated children (BELGROW). This registry collects coded data since 1985 and was approved by the Ethical Committees of the participating centres of the BESPEED members. Informed consent was secured prior to entry of data in the registry.

Inclusion criteria were as follows: (1) diagnosis of SGA (Birth weight and/or length < −2 SDS), (2) treatment with recombinant human GH (rGH), given continuously on a daily basis during at least 3 years when treatment started before puberty and at least 2 years when started near the onset of puberty, (3) breast stage <B3 for girls and a testicular volume < 10 ml for boys at the start of GH therapy, and (4) achievement of AH, defined as a height velocity < 2 cm/year. Exclusion criteria were as follows: (1) patients with a known syndrome (including Silver-Russell syndrome) and/or having major malformations and (2) treatment with GnRH agonist or other GH-promoting agents, such as oxandrolone or letrozole.

**Figure 1:**
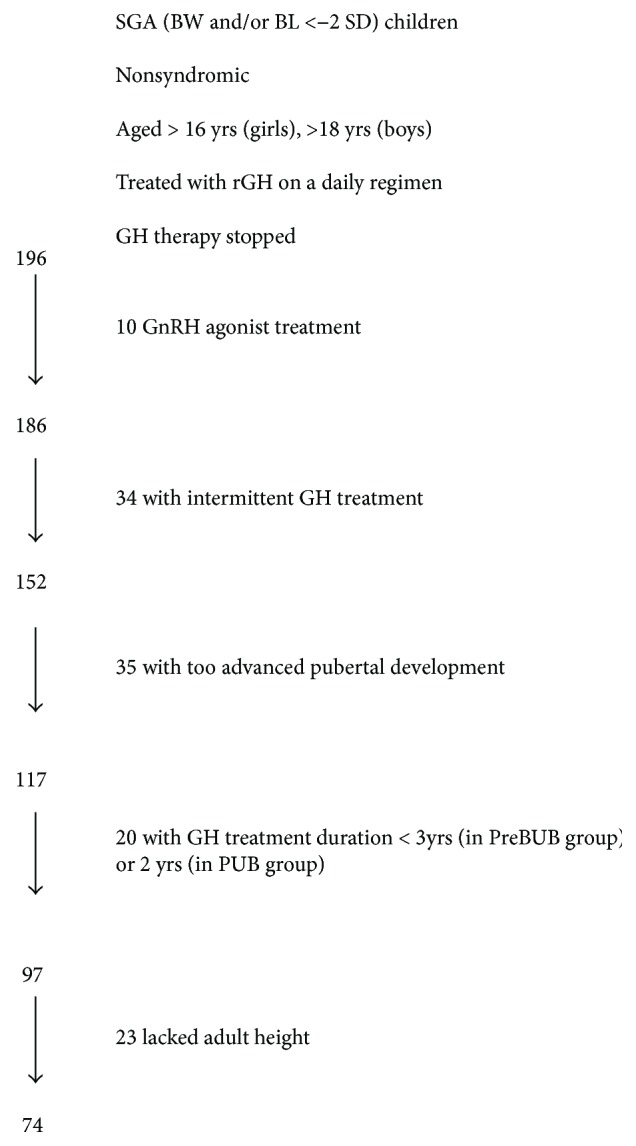
Patients selection flowchart.

In total, 196 short SGA patients treated since 1988 with a daily regimen of rGH, no documented syndrome or major malformations, aged more than 16 years (girls) and 18 years (boys) by the end of 2012, and who stopped GH therapy were retrieved from BELGROW (Figure 1). Ten patients treated with GnRH agonists were excluded, and data of 34 patients were not analysed because of intermittent GH treatment. Out of the 152 remaining patients, 35 had a too advanced pubertal development to be included. Twenty additional patients were excluded because the treatment duration was less than 3 years when prepubertal at the start of treatment or less than 2 years when pubertal at the start of treatment. For 74 (76%) of the 97 remaining patients, an AH (HV < 2 cm/year) was documented in the registry (*n* = 61) or obtained from the family doctor (*n* = 13). Birth and auxological characteristics at onset of GH treatment were comparable in patients with and without data on AH (data not shown).

The 74 included patients were divided into 2 groups according to their degree of pubertal maturation at start of GH treatment and/or during the first year of GH therapy: 27 patients (11 boys) started GH at least 1 year before the onset of puberty (PrePUB group) and 47 patients (22 boys) either started GH when they were already in puberty (at an early stage) (*n* = 28) or entered in puberty during the first year of treatment (*n* = 19) (PUB group).

### 2.2. Methods

At baseline and during follow-up visits every 3 to 6 months, the following data were collected: chronological age, height, body weight, pubertal stage, dose of GH, and adverse events. Pubertal staging was determined according to Tanner and Whitehouse [6].

Anthropometric data (height, weight, and BMI) were expressed as z-scores adjusted for age and gender using the Flemish population references [7]. AH was defined as a height reached when growth velocity was <2 cm/year. AH SDS was calculated using adult references (SDS for age 21 years). Birth weight and length were expressed as z-scores adjusted for gestational age using the reference of Niklasson et al. [8]. The midparental height (MPH) SDS was calculated as (father's height SDS + mother's height SDS)/1.61 [9]. First year height velocity and gain in height SDS were calculated if measurements were available between 9 and 15 months after the start of GH therapy. Onset of puberty was defined by a testicular volume ≥ 4 ml in boys and the presence of a breast stage 2 (B2) in girls. Pubertal height gain (cm or SDS), defined by the AH (cm or SDS) minus the height at onset of puberty (cm or SDS), was calculated if a visit was available with pubertal development B2 in girls and testis volume 4 ml in boys (*n* = 68). Total height gain was calculated as AH minus height at start of GH. The parent-specific lower limit of height SDS range was calculated as (0.5 × midparental height SDS) − 1.73 SDS [9]. Mean daily dosage (*μ*g/kg/day) during the whole treatment period was calculated using the dosage recorded at each visit.

### 2.3. Statistical Analysis

Results are expressed as mean ± SD. Both the percentage of subjects with an AH SDS > −2 and an AH SDS above the parent-specific lower limit was calculated. Continuous variables and percentages were compared across groups using unpaired *t*-tests, Mann–Whitney *U* tests, or chi-square tests as appropriate. Multiple regression analysis with backward stepwise variable selection was used to analyse the relationship between characteristics of the patients and treatment parameters as independent variables and adult height SDS or total height gain SDS as the outcome. A *p* value < 0.05 was considered statistically significant. Stata 10.1 and IBM SPSS Statistics 21® were used for the statistical analysis.

## 3. Results

### 3.1. Auxological Characteristics (Table 1)

Birth and parental auxological data were comparable in the PrePUB and PUB groups, as shown in Table 1. MPH SDS was in both groups significantly (*p* < 0.001) lower compared to the general population.

### 3.2. Growth Response, Adult Height, and Total Height Gain SDS (Table 2)

**Figure 2:**
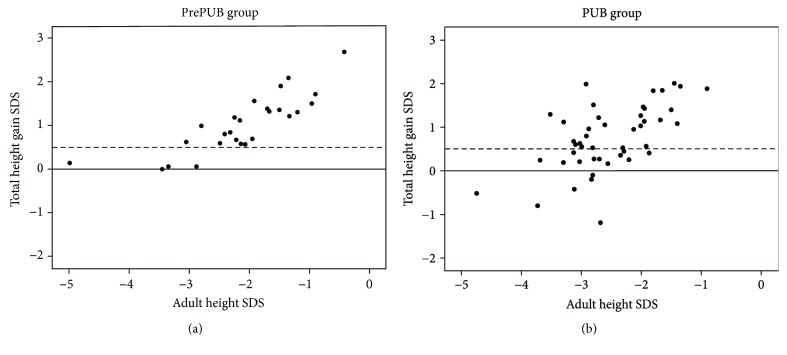
Scatterplot of total height gain SDS in relation to adult height SDS in the PrePUB group (a) and the PUB group (b).

At onset and at the end of GH treatment, height SDS was comparable in the PrePUB and PUB groups. The total height gain SDS (1.1 ± 0.7 in the PrePUB versus 0.8 ± 0.7 in the PUB group) was also similar after, respectively, 5.8 ± 2.1 and 4.1 ± 1.1 years of GH treatment (*p* < 0.001). There was no significant difference in duration of GH treatment between males and females in the PUB group, but males in the PrePUB group were treated longer (6.8 ± 2.5 versus 5.1 ± 1.3 years (*p* = 0.03)). A total height gain > 0.5 SDS was observed in 85% (23/27) of patients in the PrePUB group and in 64% (30/47) of patients in the PUB group (*p* = 0.06) (Figures 2(a) and 2(b)). Absolute AH was, respectively, 167.5 ± 7.7 in boys and 153.4 ± 4.8 cm in girls in the PrePUB group and, respectively, 165.2 ± 4.9 and 150.8 ± 4.4 cm in the PUB group. Whereas AH was above −2 SDS in 44.4% of the PrePUB group and in 27.7% of the PUB group (*p* = NS), respectively, 66.7% and 69.8% reached an AH above their parent-specific lower limit (*p* = NS).

### 3.3. Pubertal Growth (Table 3)

Height SDS at start of puberty was significantly lower in the PUB group compared to the PrePUB group. In the PUB group, there was a gain of 0.6 ± 0.7 in height SDS from onset of puberty until AH, while in the PrePUB group, height SDS increased by 0.2 ± 0.9 during this period. Total pubertal height gain in boys as well as girls was significantly higher in the PUB group than the PrePUB patients (resp., 28.2 ± 5.3 versus 23.2 ± 3.3 cm in boys and 21.5 ± 5.6 versus 16.7 ± 5.3 cm in girls).

### 3.4. Multivariate Analysis (Table 4)

Multiple linear regression was used to determine the factors influencing the AH SDS and the total height gain. The following variables were included in the model: birth length and weight SDS, gender, target height SDS, height and weight SDS at start, mean total dosage (*μ*g/kg/day), total duration of GH therapy, height gain SDS during the first year of GH therapy, and the group factor PrePUB or PUB. A significantly lower growth response and adult height outcome was observed in females in comparison with males, in patients lighter at start of GH treatment and in pubertal patients. AH SDS was positively correlated with the height SDS at start of GH.

## 4. Discussion

Our retrospective study showed that short SGA adolescents starting GH treatment just before (less than one year) the onset of puberty or at an early pubertal stage (around a mean age of 12.3 years) have a modest and variable height gain (0.8 ± 0.7 SDS) when treated for 4 years at a mean dosage of 42 *μ*g/kg/day. Only one third of them obtained a normal AH, which was positively associated with height at start. On the other hand, GH-treated SGA adolescents reached an AH within parental target height range in a similar percentage as SGA children who were treated at least 1 year before the onset of puberty with a similar dosage but for longer duration (1.7 years longer). In addition, their pubertal height gain was greater than that of the prepubertal children starting GH treatment 2.3 years earlier. Our data suggest that when short SGA adolescents are requesting GH therapy and are responding to the reimbursement criteria, they should not be excluded from GH treatment because of their older age and imminent pubertal development, but a realistic growth prognosis should always be given.

The observed pubertal height gain in the SGA subjects starting GH treatment around puberty in our study was comparable with that observed in healthy British children, which is 29.5 cm in boys (testis volume > 3 ml) and 19.2 cm in girls (Tanner stage B2) [10]. In a Spanish study, 31 untreated short SGA children had a smaller pubertal height gain than reported in the national reference population [11]. In an Israeli study, a similar total pubertal growth and peak height velocity was observed in short SGA children (*n* = 76) in comparison with short children born appropriate for gestational age (*n* = 52). However, the earlier onset of puberty in SGA children was not taken into account in this particular study [12].

In our study, age at start of puberty was relatively late, in both boys and girls. We suspect that besides the exclusion of patients with additional GnRH treatment, a recruitment bias might be involved, as mainly short adolescents with a later onset of puberty, experiencing a more pronounced prepubertal growth deceleration, might have requested GH treatment. Without GH treatment, a rather low pubertal gain had to be expected. The GH treatment allowed the short SGA patients to present with a normal pubertal height gain as in normal growing non-SGA adolescents. In the prepubertal group, the observed pubertal height gain was comparable with the increments reported by Ranke and Lindberg in a group of 59 (24 females) short SGA children treated at least two years before puberty onset [13].

Studies evaluating adult height of short SGA children starting GH around pubertal onset are scarce. Carel et al. [14] treated with GH a cohort of 91 early pubertal SGA children at a mean age of 12.6 years for a relative short duration of 2.7 ± 0.6 years, at a relatively high dosage of 67 *μ*g/kg/day. Many participants discontinued their treatment prematurely, before their height velocity was <2 cm/year. Total height gain was 1.1 ± 0.9 SDS, making it possible for 47% of the treated SGA adolescents to achieve an adult height within the normal range for the general population (>−2 SDS). Lem et al. [5] confirmed that SGA patients starting GH treatment during adolescence at a median age of 11.2 years (when 46% were already in puberty) at a dosage of either 33 or 66 *μ*g/kg/day still can have a significant catch-up growth. In 84 patients who attained AH, height improved from −2.9 SDS at start of treatment to −1.7 SDS at AH (height gain around 1.2 SDS), permitting 62% of adolescents to attain an adult height above −2 SDS.

In our study, the almost similar growth outcome of SGA children treated around puberty with those starting some years before puberty might be partly explained by the relatively short duration of GH treatment in the prepubertal group, who started GH therapy at a relatively advanced age of 10.0 years. Studies reporting on the adult height and total height gain in prepubertal short SGA children after continuous GH therapy, but starting GH treatment at a much younger mean age than our cohort, have found better growth outcomes. Mean height gain was 1.4 SDS (*n* = 162; GH start around 7.8 yr) in the study of Ranke and Lindberg [2] and 1.7 SDS (*n* = 73; GH start at 7.7 yr) in the study of Bannink et al. [1]. We expect that within the following 5 years, more SGA patients who have started GH at a younger age will attain adult height and be available in our registry for a comparative adult height analysis. In addition, the study of quality of life outcome and employment status in relation to the age at start of treatment might be of interest in this cohort of SGA patients.

Given the wide variation in GH-induced growth response in both prepubertal and pubertal subjects, predictors of the growth response and AH were evaluated. Like in other studies [2, 15, 16], AH was found to be positively related to the height SDS at start. In accordance with the findings of Dahlgren and Wikland [15] including prepubertal short SGA children, lighter SGA adolescents were found to experience better height gain in our study. We hypothesize that a lower adiposity at start of GH therapy in SGA children and especially in adolescents might induce a lesser degree of adrenal hyperandrogenism and/or compensatory hyperinsulinemia causing less bone age acceleration [17, 18]. In addition, the growth response and final height outcome to GH in our study was found to be gender dependent. The lower growth response in girls compared to boys in the PUB group was not explained by a difference in treatment duration, but may be related to the stronger influence of estrogens than androgens on bone maturation. Decreased as well as increased serum estradiol levels have been found in SGA children at completion of puberty [19]. Furthermore, SGA females, in comparison with males, might be at risk for a more pronounced adrenarche and/or insulin resistance, which are both associated with a more rapid bone maturation [20–22]. In our study, no effect of the GH dosage on AH outcome was found, but the dosage administered ranged only between 32 and 53 *μ*g/kg/day for 90% of the population. Whereas in the prepubertal years a dose-dependent height gain has been found in most studies, this might be of less importance on the long term and in pubertal SGA children [15, 23–25].

Our study has several limitations. It was a retrospective study, without a control group on a relatively small number of patients. In retrospective studies and studies in whom a great proportion are lost to follow-up (for a quarter of the patients, no adult height was reported in our registry), some overestimation of the effect might be present. However, nonregistry studies have reported similar or even better growth effects in SGA adolescents [5, 14]. On the other hand, our approach to calculate AH SDS using adult references (SDS for age 21 years) may underestimate the AH SDS as some patients could have grown a few centimetres after the last visit available in the registry. The registry does not include untreated SGA children, making a direct comparison with untreated patients not possible. In untreated short SGA adolescents, a 0.5 SDS height increase from onset of puberty has been reported previously by Carel et al. [14] and should be taken into account. Retrieval of historical controls for comparison was judged difficult from the participating centres, since longitudinal data up to adult height are needed and the secular trend might favour a higher AH in the current GH-treated group.

To increase the efficacy of GH treatment in short SGA adolescents, several options have been tried or are still under investigation. Higher GH dosages have been studied in a recent trial by Lem et al. [5]. These authors showed that SGA patients starting their treatment around puberty and treated with a GH dosage of 66 *μ*g/kg/day obtained a 0.5-0.6 SDS higher AH compared to those treated at 33 *μ*g/kg/day, after correcting for influencing variables (gender, age at start, height SDS at start, treatment years before puberty, and target height SDS). However, the decision of treating SGA patients with higher GH doses must be weighed against potential long-term safety issues, given the risk of elevated serum IGF-1 levels in up to one third of the patients when GH dosages of 66 *μ*g/kg/day are given [26]. Furthermore, the increase of GH dose is limited by the medication label for SGA.

The addition of GnRH agonists has been tested in short SGA adolescents in order to prolong GH treatment duration and improve adult height outcome [27]. There is however no convincing evidence that AH in short GH-treated SGA children can be improved by postponing pubertal onset with GnRH agonist. In a randomized study of short adolescents (Tanner stage 2 and 3), born either with appropriate Birth weight (*n* = 11) or SGA (*n* = 6) with a predicted adult height below −2 SDS and receiving GH in combination with a GnRH agonist for 3 years, no difference in adult height was observed in comparison with an age- and height-matched untreated control group (*n* = 15) [27]. Lem et al. [5] have shown that adding a GnRH agonist for 2 years in short GH-treated SGA children with a height at onset of puberty <140 cm (considered as having a poor AH expectation) had a similar AH than patients receiving GH only. Prolonging the pubertal growth phase for a much longer period by GnRH agonist and/or the addition of estrogen blocking agents should be further explored, as GnRH agonist administration for 3.5 years was found to increase adult height by 0.6 SDS in a group of 26 adolescents with very short stature of different origins [28]. The psychosocial impact of such combined treatment (school performance, social acceptance, and general self-worth) should also be further investigated.

In conclusion, our study shows that short SGA adolescents starting GH therapy at an early pubertal stage have a modest and variable height gain. The best adult height outcome can be expected in those with the lowest height deficit at start of GH treatment. Our finding that female patients and those with a higher body weight are at higher risk for a poor adult height outcome needs confirmation in larger studies.

## Figures and Tables

**Table 1:** Comparison of birth and parental auxological data between the PrePUB and PUB groups.

	*n*	PrePUB group (*n* = 27)	PUB group (*n* = 47)	Significance
Boys/girls, *n*	74	11/16	22/25	*p* = 0.61
Birth weight, SDS	73	−2.5 ± 1.0	−2.5 ± 0.8	*p* = 0.78
Birth length, SDS	71	−3.0 ± 0.9	−2.6 ± 0.8	*p* = 0.12
Father's height, SDS	67	−1.3 ± 1.1	−1.7 ± 1.0	*p* = 0.14
Mother's height, SDS	67	−1.2 ± 1.1	−1.5 ± 1.2	*p* = 0.35
Midparental height, SDS	67	−1.6 ± 1.0	−1.9 ± 0.9	*p* = 0.10

**Table 2:** Comparison of auxological data at start of GH treatment and at adult height between the PrePUB and PUB groups.

	*n*	PrePUB group		PUB group		Significance
		Males (*n* = 11)	Females (*n* = 16)	Males (*n* = 22)	Females (*n* = 25)	
*At start of GH treatment*						
Age (yr)	74	10.1 ± 3.0	10.0 ± 2.2	13.6 ± 1.3	11.3 ± 1.3	*p* < 0.001/*p* = 0.025^∗^
Height, SDS	74	−3.2 ± 0.6		−3.3 ± 0.7		*p* = 0.60
Weight, SDS	74	−3.0 ± 1.1		−2.8 ± 1.1		*p* = 0.39
BMI, SDS	73	−1.1 ± 1.1		−1.1 ± 1.2		*p* = 0.85
*At adult height*						
Age (yr)	74	18.4 ± 1.8	16.1 ± 1.4	18.7 ± 1.6	16.4 ± 1.7	*p* = 0.67/*p* = 0.65^∗^
Height, SDS	74	−2.1 ± 1.0		−2.5 ± 0.7		*p* = 0.06
Weight, SDS	67	−1.6 ± 1.1		−1.6 ± 1.0		*p* = 0.88
BMI, SDS	67	−0.6 ± 0.9		−0.3 ± 1.0		*p* = 0.39
Height SDS corr. for midparental height	67	−0.7 ± 1.1		−0.5 ± 1.0		*p* = 0.49
Height (cm)		167.5 ± 7.7	153.4 ± 4.8	165.2 ± 4.9	150.8 ± 4.4	*p* = 0.33/*p* = 0.08^∗^
Adult height > −2 SDS	74	12/27 (44.4%)		13/47 (27.7%)		*p* = 0.20
Adult height SDS > parent-specific lower limit of height	67	16/24 (66.7%)		30/43 (69.8%)		*p* = 0.79
Height gain, SDS	74	1.1 ± 0.7		0.8 ± 0.7		*p* = 0.09
Daily GH dosage (*μ*g/kg/day)	74	45.2 ± 8.5		41.8 ± 8.4		*p* = 0.09
Duration GH therapy (yr)	74	6.8 ± 2.5	5.1 ± 1.3	4.1 ± 1.1	4.1 ± 1.2	*p* < 0.001/*p* = 0.02^∗^

^∗^In males from the PrePUB group versus the PUB group/in females from the PrePUB group versus the PUB group.

**Table 3:** Comparison of auxological data at onset of puberty and during puberty between the PrePUB and PUB groups.

	*n*	PrePUB group			PUB group			Significance
		*n*	Males (*n* = 11)	Females (*n* = 16)	*n*	Males (*n* = 22)	Females (*n* = 25)	
*Height gain from start of GH until* *pubertal onset*
Delta height, SDS	53	26	0.9 ± 0.6		27^∗∗^	0.2 ± 0.2		*p* < 0.001
*Data at start of puberty*
Age (yr)	68	26	13.4 ± 1.3	12.1 ± 2.0	42	13.4 ± 1.1	11.6 ± 1.2	*p* = 0.98/*p* = 0.25^∗^
Height, SDS	68	26	−2.3 ± 0.6		42	−3.1 ± 0.7		*p* < 0.001
Weight, SDS	68	26	−2.2 ± 1.0		42	−2.7 ± 1.1		*p* = 0.04
BMI, SDS	68	26	−1.0 ± 0.9		42	−1.2 ± 1.2		*p* = 0.68
*Height gain from onset of puberty* *until AH*
Delta height, SDS	68	26	0.2 ± 0.9		42	0.6 ± 0.7		*p* = 0.056
Delta height (cm)	68	26	23.2 ± 3.3	16.7 ± 5.3	42	28.2 ± 5.3	21.5 ± 5.6	*p* = 0.01/*p* = 0.01^∗^

^∗^In males from the PrePUB group versus the PUB group/in females from the PrePUB group versus the PUB group. ^∗∗^In the PUB group, height gain from start of GH until pubertal onset was calculated only if GH therapy was started before or at onset of puberty.

**Table 4:** Results of multiple linear regression analysis with adult height SDS and total height gain SDS as dependent variables (2 groups combined).

	Adult height SDS		Total height gain SDS	
	*R*	*p*	*R*	*p*
Intercept	0.97		1.46	
Gender (female)	−0.33	0.042	−0.34	0.04
Height SDS at start	0.89	<0.001	—	
Weight SDS at start	−0.18	0.040	−0.21	0.006
Mean GH dosage (*μ*g/kg/day)	−0.02	0.081	−0.02	0.06
Group (pubertal)	−0.35	0.042	−0.33	0.05
*R* ^2^	0.43		0.21	
